# Inorganic Polyphosphate Modulates TRPM8 Channels

**DOI:** 10.1371/journal.pone.0005404

**Published:** 2009-04-30

**Authors:** Eleonora Zakharian, Baskaran Thyagarajan, Robert J. French, Evgeny Pavlov, Tibor Rohacs

**Affiliations:** 1 Department of Pharmacology and Physiology, University of Medicine and Dentistry of New Jersey-New Jersey Medical School, Newark, New Jersey, United States of America; 2 Department of Physiology and Biophysics, University of Calgary, Calgary, Alberta, Canada; Duke Unviersity, United States of America

## Abstract

Polyphosphate (polyP) is an inorganic polymer built of tens to hundreds of phosphates, linked by high-energy phosphoanhydride bonds. PolyP forms complexes and modulates activities of many proteins including ion channels. Here we investigated the role of polyP in the function of the transient receptor potential melastatin 8 (TRPM8) channel. Using whole-cell patch-clamp and fluorescent calcium measurements we demonstrate that enzymatic breakdown of polyP by exopolyphosphatase (scPPX1) inhibits channel activity in human embryonic kidney and F-11 neuronal cells expressing TRPM8. We demonstrate that the TRPM8 channel protein is associated with polyP. Furthermore, addition of scPPX1 altered the voltage-dependence and blocked the activity of the purified TRPM8 channels reconstituted into planar lipid bilayers, where the activity of the channel was initiated by cold and menthol in the presence of phosphatidylinositol 4,5-biphosphate (PtdIns(4,5)P_2_). The biochemical analysis of the TRPM8 protein also uncovered the presence of poly-(R)-3-hydroxybutyrate (PHB), which is frequently associated with polyP. We conclude that the TRPM8 protein forms a stable complex with polyP and its presence is essential for normal channel activity.

## Introduction

TRPM8 is a member of the transient receptor potential (TRP) channel family of the melastatin subgroup, which is thought to be a major sensor for a wide range of cold temperatures in the peripheral nervous system [1], [2], [3]. TRPM8 is activated by low temperatures in the range of 8–26°C and a number of chemical compounds such as menthol, icilin, eucalyptol, geraniol and linalool [4], [5], [6]. Several other factors, such as voltage [7], [8], pH [8], lysophospholipids and fatty acids [9], [10] also modulate TRPM8 activity.

A major intracellular factor that is required for the channels activity of TRPM8 is phosphatidylinositol 4,5-biphosphate (PtdIns(4,5)P_2_) [11], [12]. PtdIns(4,5)P_2_ regulation is a common property of many TRP channels [13], [14], [15] and several other ion channels from different families [16], [17], [18], [19]. In general the dynamic changes in the levels of plasma membrane phosphoinositides have been shown to play regulatory roles in many ion transporting systems [20], [21], [22]. TRP channel functions could also be modified by inorganic polyphosphates apart from phosphoinositides. Recently it has been shown that TRPA1 channels can be activated by pungent chemicals only in the presence of inorganic polyphosphates [23].

Inorganic polyphosphate (poly P) is a polymer of tens or hundreds of phosphate residues linked by high-energy anhydride bonds as in ATP. PolyP plays central roles in many general physiological processes, acting as a reservoir of energy and phosphate, as a chelator of metals, as a buffer against alkali. In microorganisms it is essential, for example, for physiological adjustments to growth conditions as well as to stress response [24]. Polyphosphates are present in all higher eukaryotic organisms, where they likely play multiple important roles [25], [26], [27]. In higher eukaryotes, polyP contributes to the stimulation of mammalian target of rapamycin, involved in the proliferation of mammary cancer cells [28] and regulates mitochondrial function [29]. However, many aspects of polyP function in these organisms remain to be uncovered.

PolyP is also believed to be an important participant in ion transport. PolyP, in association with a solvating amphiphilic polymer of R-3-hydroxybutyrate (PHB), can form ion channels with high selectivity for cations [30]. Channel forming polyP/PHB Ca^2+^ complexes have been found in bacterial and mitochondrial membranes [30], [31], [32]. Furthermore, polyP and PHB are associated with a variety of membrane proteins, including several bacterial ion channels and might be required for their normal functioning [33], [34].

In the present study, we demonstrate that TRPM8 expressed in HEK-293 and F-11 neuronal cells is associated with polyP and PHB, and that polyP serves as crucial regulator of TRPM8 channel function.

## Methods

### Cell culture

HEK-293 cells were maintained in minimal essential medium (MEM) solution (Invitrogen, San Diego, CA) supplemented with 10% fetal bovine serum (Invitrogen) and 1% penicillin/streptomycin. The rat TRPM8 tagged with the *myc* epitope on the N-terminus, scPPX1, GFP in pCDNA3 vectors were transfected using the Effectene reagent (Qiagen, Chatsworth, CA). Two different TRPM8 stable cell lines were developed: one with TRPM8 *myc*-tagged on the N-terminus (TRPM8-*myc*), and one with TRPM8 tagged with *myc* on the N-terminus and with 6His residues on the C-terminus (TRPM8-*his*). These stable cell lines were obtained using the following procedure: HEK-293 cells were treated with different concentration of G418 to determine killing concentration of G418 (Sigma, St. Louis, MO). Then cells were transfeced with lineralized TRPM8-myc or TRPM8-his cDNA using effectene transfection reagent. 24 hours after transfection, cells were treated with 1 mg/ml G418 containing MEM supplemented with 10% FBS and antibiotics. After 7 days, single cells were selected from clonal rings and these were seeded on 24 well plates for further propagation of each single clone. The individual clones were pooled into a single culture and propagated in the presence of 400 µg/ml G418. Forty eight hrs before the experiment, cells were split into MEM supplemented with FBS and antibiotics but without G418.

F-11 cells were cultured in DMEM/F12 medium +20% FBS, 0.2 mM L-glutamine, 100 µM sodium hypoxanthine, 400 nM aminopterin, 16 µM thymidine (HAT supplement), and penicillin/streptomycin at 37°C (the cells were kindly provided by Dr. S.E. Gordon, University of Washington).

### Mammalian electrophysiology

Whole-cell patch clamp measurements were conducted 36–72 h after propagation of the TRPM8 stable cell lines or transient transfection of target clones. The extracellular solution contained (in mM) 137 NaCl, 5 KCl, 1 MgCl_2_, 10 glucose, and 10 HEPES, pH 7.4. Borosilicate glass pipettes (World Precision Instruments, Sarasota, FL) of 2–4 MΩ resistance were filled with a solution containing (in mM) 135 K-gluconate, 5 KCl, 5 EGTA, 1 MgCl_2_, and 10 HEPES, pH 7.2. For the experiments the pipette solution was supplemented with 2 mM ATP. After formation of GΩ-resistance seals, whole-cell configuration was established, and currents were measured at a holding potential of −60 mV, using an Axopatch 200B amplifier (Molecular Devices, Union City, CA). Current-voltage ramp relations were recorded using voltage ramps from −100 to +100 mV with a duratron of 0.8 s. Data were collected and analyzed with the pClamp 9.0 software. Measurements were performed at room temperature (∼22°C).

### Intracellular Ca^2+^ measurements

The extracellular solution used in ratio-metric [Ca^2+^]_i_ measurements contained (in mM) 137 NaCl, 5 KCl, 1.8 CaCl_2_, 1 MgCl_2_, 10 Glucose and 10 Hepes, pH 7.4. Cells were incubated with 2 µM Fura-2 acetoxymethyl ester (Tef Labs Austin, TX) for 30 min. at room temperature. The fluorescence signals of single cells were measured using alternating excitation at 340 and 380 nm and emission was detected at 510 nm. The ratio of fluorescence (340/380) was plotted against time. The measurements were performed using a Photon Technology International (PTI) (Birmingham, NJ) photomultiplier based system mounted on an Olympus IX71 microscope, equipped with a DeltaRAM excitation light source, or with a Ratiomaster 5 Imaging System (PTI) equipped with a Cool-snap HQ2 (Roper) Camera.

### Preparation of the TRPM8 protein

HEK-293 cells stably expressing TRPM8 were grown to 70–80% confluence, washed and collected with cold PBS. Cells were harvested and resuspended in 0.25 M sucrose-1 mM triethanolamine (TEA) HCl, with addition of a protease inhibitor cocktail (Roche, Indianapolis, IN), pH 7.4. Plasma membranes were isolated by differential centrifugation. The TRPM8 protein was extracted from plasma membranes with (in mM) 137 NaCl, 5 KCl, 1 MgCl_2_, 10 Glucose and 10 Hepes, pH 7.4, in presence of 1% Nonidet P40 (Roche) and 0.5% dodecyl-maltoside (DDM) (Roche), and the protease inhibitors, upon incubation at 4°C on a shaker with gentle agitation for 2 h. This suspension was further centrifuged for 1 h. at 100,000 g. The supernatant was concentrated with 100 K Amicon centrifuge filters (Millipore-Fisher) and purified by gel-filtration chromatography on Sephacryl S-300 column (1.6×60 cm GE Healthcare, Piscataway, NJ) equilibrated with the same buffer containing 2 mM DDM. All steps of purification were performed at 4°C. After elution from the column, protein fractions were concentrated to a final concentration of 12 µg/ml and analyzed by Western blot analysis with anti-c-Myc IgG antibodies (Sigma). For some of the planar lipid bilayer experiments, in order to improve the stability of the artificial membranes with the incorporated protein, we also purified TRPM8 from the TRPM8-*his* stable cell line. This modification allowed us to include into the procedure described above an additional step of purification with ion-affinity chromatography using Ni-NTA beads (Qiagen).

### SDS-PAGE

Proteins were electrophoretically separated on 7.5 or 10% SDS-PAGE (Bio-Rad, Hercules, CA) using Tris-glycine sodium dodecyl sulfate (SDS) buffer (Bio-Rad) at a constant voltage of 180 V. The electrophoresis buffer for the native gels did not contain SDS. Protein bands were visualized by staining with Coomassie brilliant blue R-250. For Western blot analysis, protein was transferred onto nitrocellulose membranes (Bio-Rad) in 10 mM CAPS, 0.07% SDS buffer at 30 V overnight. The TRPM8 protein was detected with anti-Myc-IgG antibodies.

### Determination of polyP

PolyP was visualized on the native 7.5% or 10% polyacrylamide Ready Gels from Bio-Rad (Helcules, CA, USA). Electrophoresis was performed at 100 V for 1–1.5 h. Gels were incubated for 1 h. in fixative solution consisting of 25% methanol / 5% glycerol, stained for 30 min with 0.05% -*o*-toluidine blue and destained in a fixative for 2 hours. To eliminate polyP, the samples were treated with 2 µg/ml exopolyphosphatase of *Saccharomyces cerevisiae* scPPX1.

### Determination of PHB

PHB was detected by Western blot analysis with anti-PHB IgG raised in rabbits to a synthetic 8-mer of R-3-hydroxybutyrate (kindly provided by Dr. Rosetta N. Reusch).

### Planar lipid bilayer measurements

Planar lipid bilayers were formed from a solution of synthetic 1-palmitoyl-2-oleoyl-glycero-3-phosphocoline (POPC) and 1-palmitoyl-2-oleoyl-glycero-3-phosphoethanolaminein (POPE, Avanti Polar Lipids, Birmingham, AL) in ratio 3∶1 in n-decane (Aldrich). The solution was used to paint a bilayer in an aperture of ∼150 µm diameter in a Delrin cup (Warner Instruments, Hamden, CT) between symmetric aqueous bathing solutions of 150 mM KCl, 20 mM Hepes, pH 7.2, at 22°C. All salts were ultrapure (>99%) (Aldrich). Bilayer capacitances were in the range of 50–75 pF. After the bilayers were formed, 0.2–0.5 µl of the TRPM8 micellar solution (2 µg/ml) was added to the *cis* compartment with gentle stirring. Unitary currents were recorded with an integrating patch clamp amplifier (Axopatch 200A, Axon Instruments). The *trans* solution (voltage command side) was connected to the CV 201A head stage input, and the *cis* solution was held at virtual ground *via* a pair of matched Ag-AgCl electrodes. Currents through the voltage-clamped bilayers (background conductance <3 pS) were filtered at the amplifier output (low pass, −3 dB at 10 kHz, 8-pole Bessel response). Data were secondarily filtered at 50 Hz through an 8-pole Bessel filter (950 TAF, Frequency Devices) and digitized at 1 kHz using an analog-to-digital converter (Digidata 1322A, Axon Instruments), controlled by pClamp9 software (Axon Instruments). Single-channel conductance events, all points' histograms, open probabilities and other parameters were identified and analyzed using the Clampfit9 software (Axon Instruments).

### Temperature Studies

For temperature studies, a Delrin cuvette was seated in a bilayer recording chamber made of a thermally conductive plastic (Warner Instruments). The chamber was fitted on a conductive stage containing a pyroelectric heater/cooler. Deionized water was circulated through this stage, pumped into the system to remove the heat generated. The pyroelectric heating/cooling stage was driven by a temperature controller (CL-100, Warner Instruments). The temperature of the bath was monitored constantly with a thermoelectric device in the *cis* side, i.e. the ground side of the cuvette. Although there was a temperature gradient between the bath solution and conductive stage, the temperature within the bath could be reliably controlled within ±0.5°C.

## Results

### Inhibition of TRPM8 currents and Ca^2+^ signals by exopolyphophatase

**Figure 1:**
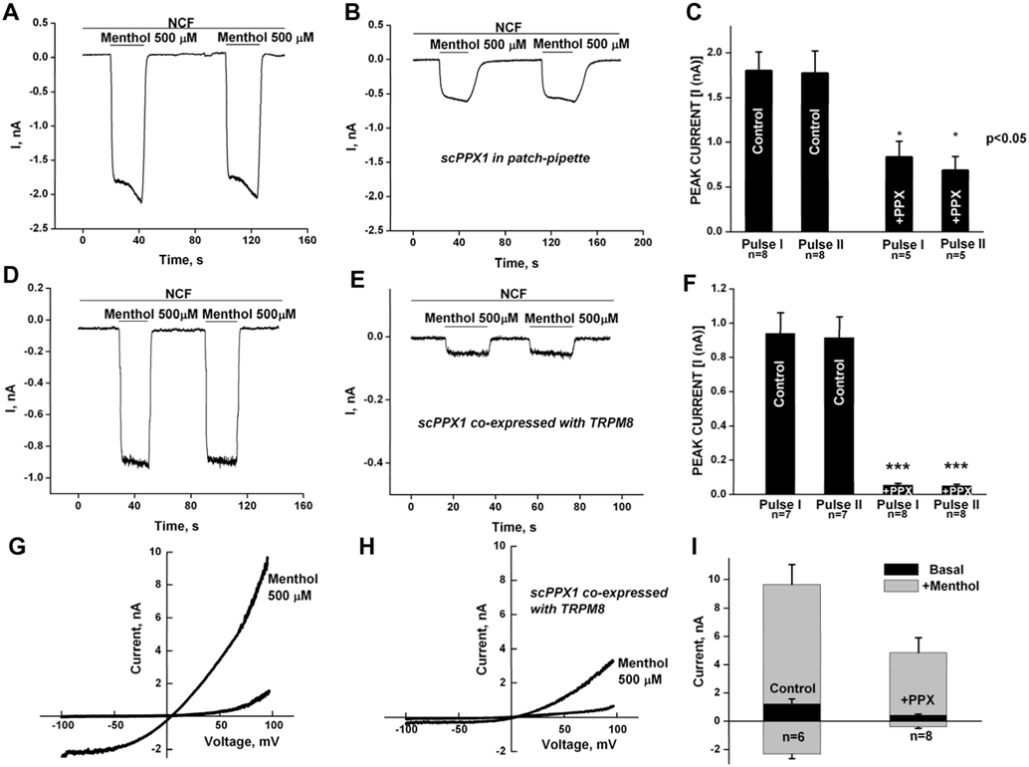
Inhibition of TRPM8 currents by scPPX1 in whole-cell patch clamp. Upper panels: Whole-cell patch clamp measurements of menthol-induced currents were performed at −60 mV in the whole-cell configuration on HEK cells expressing TRPM8, in nominally Ca^2+^-free solution (NCF), to avoid desensitization. Menthol pulses (500 µM) were applied in the first 3–5 min after establishment of whole-cell configuration: HEK-293 cells were transiently transfected with TRPM8 (0.4 µg) and co-transfected with GFP clone (0.2 µg) to allow detection of transfected cells. Panel A: the control. Panel B: the pipette solution was supplemented with 2.3 µg/ml scPPX1. Midle panels: Whole-cell patch clamp was performed on HEK-293 TRPM8 stable cell line, which was transiently transfected with GFP (0.2 µg) alone (panel D) or with scPPX1 clone (0.4 µg) and GFP (0.2 µg) (panel E). The summaries are shown in panel F. The protocol of experiment is the same as for the measurements in the upper panel. Lower panels: Current/Voltage relationships of TRPM8 channels obtained in whole-cell patch clamp performed at −100 +100 mV voltage ramps for HEK-293 TRPM8 stable cell line, which was transiently transfected with GFP (0.2 µg) alone (panel G) or with scPPX1 clone (0.4 µg) and GFP (0.2 µg) (panel H). The summaries are shown in panel I at −100 and +100 mV.

In developing the methods for our studies we have used an enzymatic approach with application of *Saccharomyces cerevisiae* exopolyphosphatase X (scPPX1). ScPPX1 is an effective polyphosphatase that possesses high substrate specificity and hydrolyses orthophosphates from polyP chains of varied lengths, but not from ATP, pyrophosphate and trimetaphosphate [35]. In order to detect a possible effect of polyP on TRPM8 we conducted a number of experiments with application, or expression, of scPPX1 in HEK-293 cells. In whole-cell patch clamp experiments, dialysis of purified scPPX1 through the patch pipette into cells transiently transfected with TRPM8 significantly inhibited menthol-induced currents in a time period of 3–5 min of treatment with scPPX1 (Fig. 1A–C). The concentration of scPPX1 in the pipette solution (2.3 µg/ml) was sufficient to observe inhibition within the tested time. We found that higher concentrations of scPPX1 were toxic for the cells. In control experiments the menthol-activated TRPM8 currents were found to be 1.8±0.21 and 1.77±0.25 nA (n = 8) for the first and the second pulses of menthol application, while the values were found to be 0.83±0.18 and 0.68±0.16 nA (n = 5) in scPPX1 dialyzed cells. The recordings were obtained at holding potential of −60 mV. The results are summarized in figure 1C. All the errors are expressed as SEM.

We next tested the effect of scPPX1 by transiently transfecting scPPX1-pcDNA (0.4 µg) into HEK-293 cells stably expressing TRPM8 (TRPM8-HEK293). Cells were co-transfected with GFP (0.2 µg) to allow detection of transfected cells (Fig. 1D–F). Control experiments were performed in TRPM8-HEK293 cells expressing GFP alone. In controls, the values of menthol-induced currents obtained at −60 mV were 0.94±0.12 and 0.915±0.122 nA (n = 7) and in scPPX1 expressing cells the values were found to be 0.054±0.001 and 0.051±0.008 nA (n = 8), for the first and the second pulses, respectively.

The current-voltage relationships of TRPM8 channels in the control cells and scPPX1-expressing cells are demonstrated in Figure 1G–I. We found that inward currents of TRPM8 exhibit more profound inhibition by scPPX1 (∼83%) than outward currents (∼65%).

**Figure 2:**
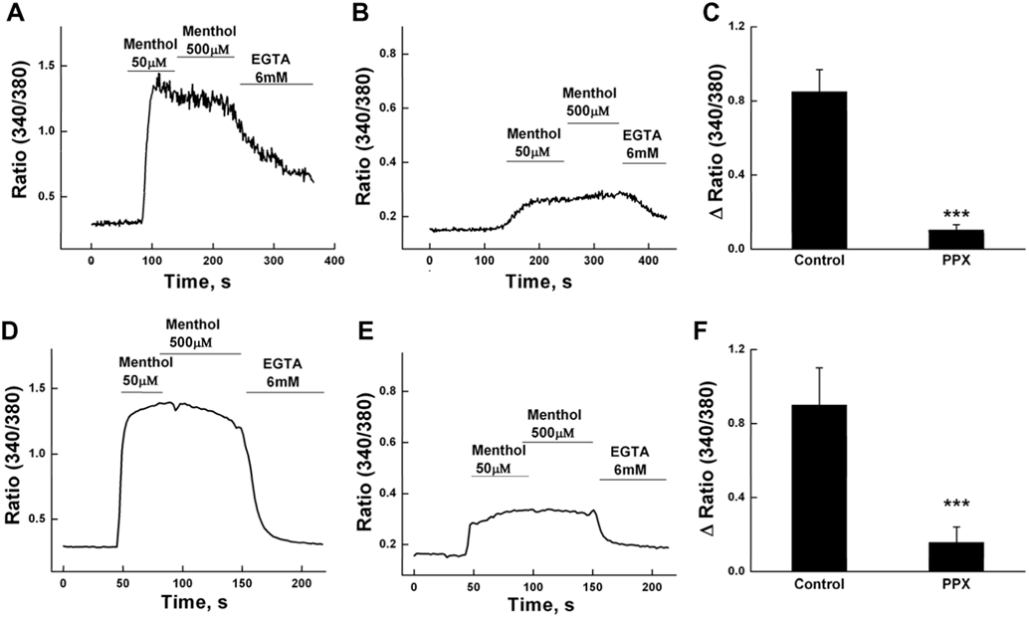
Inhibition of TRPM8 activity by scPPX1 in intracellular Ca^2+^ measurements. Upper panels: Fluorescence measurements of intracellular Ca^2+^ concentration were performed on HEK-293 TRPM8 stable cell lines with transiently transfected GFP (0.2 µg) alone (panel A) or together with the scPPX1 clone (0.4 µg) (panel B). The summaries of averaged menthol responses are represented in panel C. Lower panels: Fluorescence measurements of intracellular Ca^2+^ signals were performed on F-11 neuronal cells with transiently transfected TRPM8 (0.4 µg) and GFP (0.2 µg) (panel D) or together with the scPPX1 clone (0.4 µg) (panel E). The summaries of averaged menthol responses are represented in panel F.

Next we monitored intracellular Ca^2+^ signals induced by menthol in single TRPM8-HEK293 cells co-expressing scPPX1. Figure 2 (A–C) shows representative experiments, where 50 and 500 µM menthol were added to single cells in the presence of 1.8 mM Ca^2+^. Menthol-evoked Ca^2+^ signals were observed as an increase in the fluorescence intensity ratio of fura-2 (340/380). We found that menthol-induced intracellular Ca^2+^ signals were significantly inhibited (p≤0.005) in cells with co-expressed scPPX1 (0.156±0.085, n = 9) in comparison to the control cells (0.9±0.2, n = 6) (Fig. 2A–C). Further we performed analogous measurements in F-11 neuronal cells that were derived from rat dorsal root ganglion neurons. F-11 cells are used as a model for DRG neurons to study sensory TRP channels in a system resembling their native environment [36]. In our experiments, F-11 cells were transiently transfected with TRPM8 (0.4 µg) alone or together with scPPX1 (0.4 µg), and menthol-induced Ca^2+^ signals were subsequently analyzed (Fig. 2D–F). Similarly to the HEK cells expression system, we found that menthol-evoked Ca^2+^-entry was significantly inhibited (p≤0.005) in F-11 cells with co-expressed scPPX1 (0.105±0.029, n = 12) in comparison to the control cells expressing TRPM8 channels alone (0.85±0.119, n = 11) (Fig. 2D–F).

In order to test whether the significant inhibition of TRPM8 channel activity by scPPX1 detected in the patch clamp and Ca^2+^ measurements is due to the alteration of the levels and/or localization of the TRPM8 protein, we performed Western blot and immunocytochemical analyses on HEK-293 cells expressing TRPM8 alone, or with the enzyme (see Methods S1). In the immunocytochemistry experiments, TRPM8 showed both intracellular and plasma membrane localization, consistent with earlier studies [37]. Co-expression of scPPX1 did not alter the localization of TRPM8 (Fig. S1A) and did not decrease the amount of the protein as detected with Western blot (Fig. S1B).

TRPM8 channels require PtdIns(4,5)P_2_ for activity. In order to ensure that expression of scPPX1 did not affect PtdIns(4,5)P_2_ levels in the cells, we have monitored the distribution of the GFP-tagged PH-domain of phospholipase C δ1 in control HEK cells, and in cells transfected with scPPX1. Co-expression of scPPX1 did not change the plasma membrane localization of the GFP tagged PH domain, indicating that plasma membrane PtdIns(4,5)P_2_ levels were not significantly altered (data not shown).

### Inorganic polyphosphate and polyhydroxybutyrate associate with TRPM8

**Figure 3:**
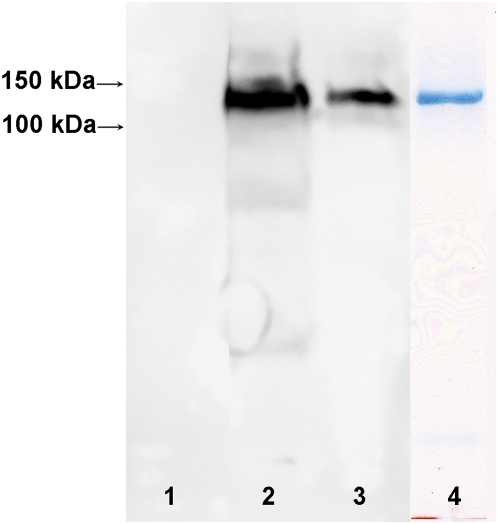
Western blots of TRPM8 protein derived from expression in HEK-293 cell lines. TRPM8 protein samples were separated on a 10% SDS-PAGE and blotted on nitrocellulose membranes overnight in the presence of CAPS buffer (pH 11.1). Immunodetection was revealed by chemiluminescence. Lanes 1–3 probed with anti-Myc-IgG: Lane 1 – plasma membrane fractions of HEK-293 cells not expressing TRPM8; Lane 2 – plasma membrane extracts of cells stably expressing TRPM8; Lane 3 – TRPM8 protein purified on Sephacryl-300 gel-filtration chromatography. Lane 4 – Coomassie blue staining of purified TRPM8. Samples were heated for 5 min. at 70°C before loading.

The dramatic inhibition of TRPM8 channel activity by scPPX1 led us to investigate whether polyP is associated with the protein. For our studies of the biochemical and biophysical properties of TRPM8, we purified the protein from the HEK-293 cell line stably expressing TRPM8. Plasma membranes were isolated by differential centrifugation with subsequent extraction of the TRPM8 protein with 1% Nonidet and 0.5% dodecylmaltoside (DDM) (see materials and methods). These conditions were favorable for harvesting a large amount of TRPM8 from the plasma membranes of cells stably expressing the protein. For control, the same extraction conditions were applied to the plasma membranes of HEK-293 cells not expressing TRPM8, where no TRPM8 protein was detected with anti-Myc IgG (Fig. 3, lane 1). In order to receive homogeneous fraction of the protein, TRPM8 was further purified by gel-filtration chromatography on Sehpacryl-300 column in 134 NaCl mM, 5 KCl mM, 1 MgCl_2_ mM and 10 Hepes mM, pH 7.4, containing 2 mM DDM. After elution from the column, fractions of the protein were concentrated in amicon-100 centrifuge tubes and analyzed by Western blot with anti-Myc IgG (Fig. 3). Analogously, we have tested a number of other detergents, including decylmaltoside, LDAO, octylglucoside, triton-X100, etc. for TRPM8 extraction and purification purposes, however only DDM resulted in a relatively high yield of purified protein and supported the stability of a tetrameric form of TRPM8, which was identified by gel-filtration chromatography and electrophoresis on the native gels.

**Figure 4:**
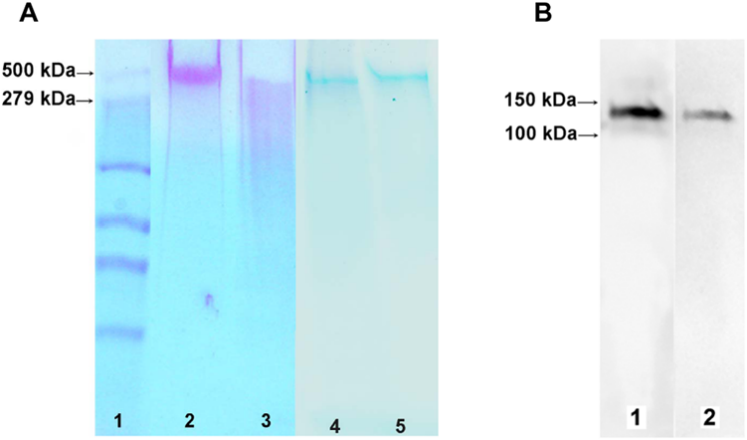
A. Detection of polyP associated with the TRPM8 protein. TRPM8 was separated on native PAGE to preserve its migration in the tetrameric form. Lane 1 – standards ladder (The High-Mark Pre-stained High Molecular Weight Protein Standards, Invitrogen); Lane 2 – purified TRPM8 sample with *o*-toluidine blue stain of native PAGE gel; Lane 3 – *o*-toluidine blue stain of native PAGE gel of the same TRPM8 sample treated with 1 µl scPPX1 (2 µg/ml) for 3 h. before loading: Lane 4 and 5 are lanes 2 and 3 re-stained with Coomassie blue. B. Detection of PHB in TRPM8 in Western blot. Lane 1 – purified TRPM8 protein detected with antiMyc_IgG; Lane 2 – Western blot of purified TRPM8 probed with anti-PHB-IgG. Samples were heated for 5 min. at 70°C before loading.

The presence of polyP in tetramers of TRPM8 was detected by its metachromatic reaction to the cationic dye, *o*-toluidine blue. PolyP of >5 residues causes a shift in the absorption on maximum of *o*-toluidine blue toward shorter wavelengths, i.e., from 630 nm (blue) to 530 nm (violet-red) [38]. PolyP stains a distinctive reddish-purple color on PAGE gels (Fig. 4A, lane 2). The identity of polyP was confirmed by its complete degradation when TRPM8 was incubated with 2 µg/ml scPPX1 (Wurst et al., 1995) for 3 h at 37°C before loading on the gel (Fig. 4A, lane 3). The presence of TRPM8 in lanes 2 and 3 of the gel was confirmed by re-staining the gel with Coomassie blue (lanes 4 and 5). The protein and polyP detected on the native gels migrate at an apparent molecular weight of 490–500 kDa, which corresponds to the molecular weight of TRPM8 in the tetrameric form. The association of polyP with the TRPM8 protein was confirmed after each protein purification procedure. A total of 12 native PAGE experiments were performed for detection of polyP.

Association of polyP with proteins has frequently been found to be mediated by PHB, which is known to “solvate” metal cation salts of polyP [30], [39], [40]. We observed that PHB was also associated with TRPM8, which was detected by Western Blot analysis using anti-PHB IgG [41] raised in rabbits to a synthetic 8-mer of R-3-hydroxybutyrate (Fig. 4B, lane 2).

### Inhibition of TRPM8 channel activity by scPPX1 in Planar Lipid Bilayers

The whole cell patch clamp experiments and intracellular Ca^2+^ measurements demonstrated that depletion of polyP by the exopolyphosphatase scPPX1 inhibited TRPM8 currents and Ca^2+^-entry. To understand whether the effect of polyP is direct or indirect on the TRPM8 channel protein, we examined the single channel properties of TRPM8 incorporated in planar lipid bilayers and the effect of subsequent treatment of the protein with scPPX1. The purified TRPM8 protein derived in dodecylmaltoside (DDM) micelles was incorporated into lipid micelles consisting of a mixture of 1-palmitoyl-2-oleoyl-glycero-3-phosphocoline and 1-palmitoyl-2-oleoyl-glycero-3-phosphoethanolaminein POPC/POPE (3∶1, v/v), and then into planar lipid bilayers of the same lipid composition between aqueous solutions of 150 mM KCl, 0.2 mM MgCl_2_ in 20 mM Hepes, pH 7.2. The presence of Mg^2+^ in the experimental solution was required to sustain normal channel activity of TRPM8 with optimal concentration of 0.2 mM. Higher concentrations of Mg^2+^ (≥2 mM) evoked an inhibition of TRPM8 currents. We also found that the presence of Mg^2+^ was necessary during the protein purification. In the absence of this cation the tetramers of TRPM8 would disintegrate into the monomers, and that in its turn would cause polyP dissociation. To confirm the stability of TRPM8 in tetrameric form and the presence of polyP the native PAGE were performed after each protein purification.

**Figure 5:**
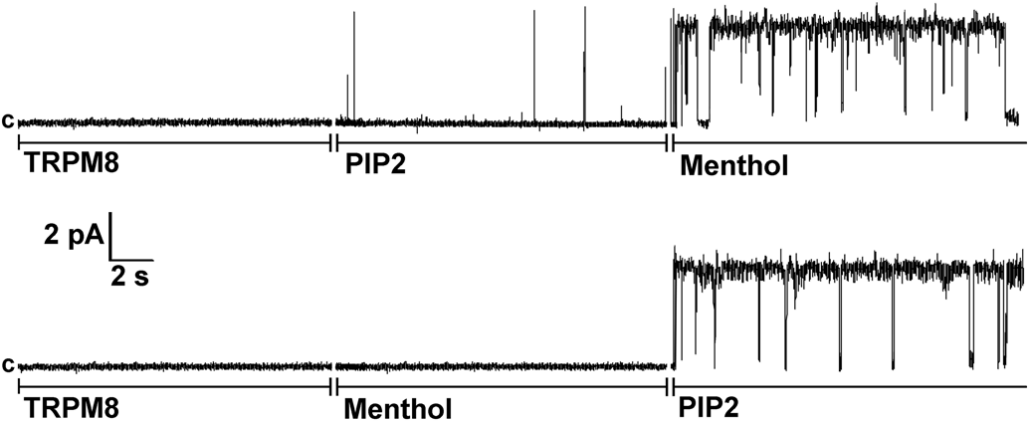
Activation of TRPM8 channels in Planar Lipid Bilayers by menthol and PtdIns(4,5)P_2_. Representative single-channel current recordings of TRPM8 channels incorporated in planar lipid bilayers formed from POPC/POPE (3∶1) in *n*-decane, between symmetric bathing solutions of 150 mM KCl, 0.2 mM MgCl_2_ in 20 mM Hepes buffer, pH 7.4 at 22°C. 0.2–0.5 µl of 0.2 µg/ml TRPM8 protein (isolated from the plasma membrane of HEK-293 cells stably expressing TRPM8) was incorporated in POPC/POPE micelles, which were added to the *cis* compartment (ground). Clamping potential was +60 mV. Data were filtered at 50 Hz. Upper and lower traces consist of three segments with additions of components as indicated in the figure: 2 µM of diC_8_ PtdIns(4,5)P_2_ and 500 µM of menthol were added to both compartments. The current recordings are representative of a total of 22 independent experiments for the upper traces and 12 independent experiments for the lower traces.

In order to stimulate channel activity we supplemented the experimental conditions with menthol and/or PtdIns(4,5)P_2_. All experiments were conducted at room temperature (∼22°C). The representative current traces of TRPM8 channels in planar lipid bilayers are given in Figure 5. No channels were observed when TRPM8 alone was incorporated in the lipid bilayers (Fig. 5, n = 13). However, addition of 2 µM of the short acyl-chain dioctanoyl (diC_8_) PtdIns(4,5)P_2_ resulted in rare burst openings of TRPM8 (P_o_<0.001, n = 12), which was followed by full opening of the channels upon addition of 500 µM menthol (P_o_ = 0.9±0.1, n = 11) (Fig. 5, upper trace). No TRPM8 openings were detected when menthol was added first, and fully open channels were observed when 2 µM diC_8_ PtdIns(4,5)P_2_ was supplemented into the bilayer (P_o_ = 0.9±0.1, n = 17) (Fig. 5, lower trace).

All the following bilayer experiments were conducted in presence of 1% 1,2-dipalmitoyl (diC_16_) PtdIns(4,5)P_2_, which resulted in higher stability of the planar lipid bilayers in comparison to the short chain diC_8_ PtdIns(4,5)P_2_. No menthol-activated channels were observed in presence of PtdIns(4,5)P_2_ on plasma membrane fractions from HEK-293 cells not expressing TRPM8, total 11 experiments were conducted from three different plasma membrane preparations (data not shown).

**Figure 6:**
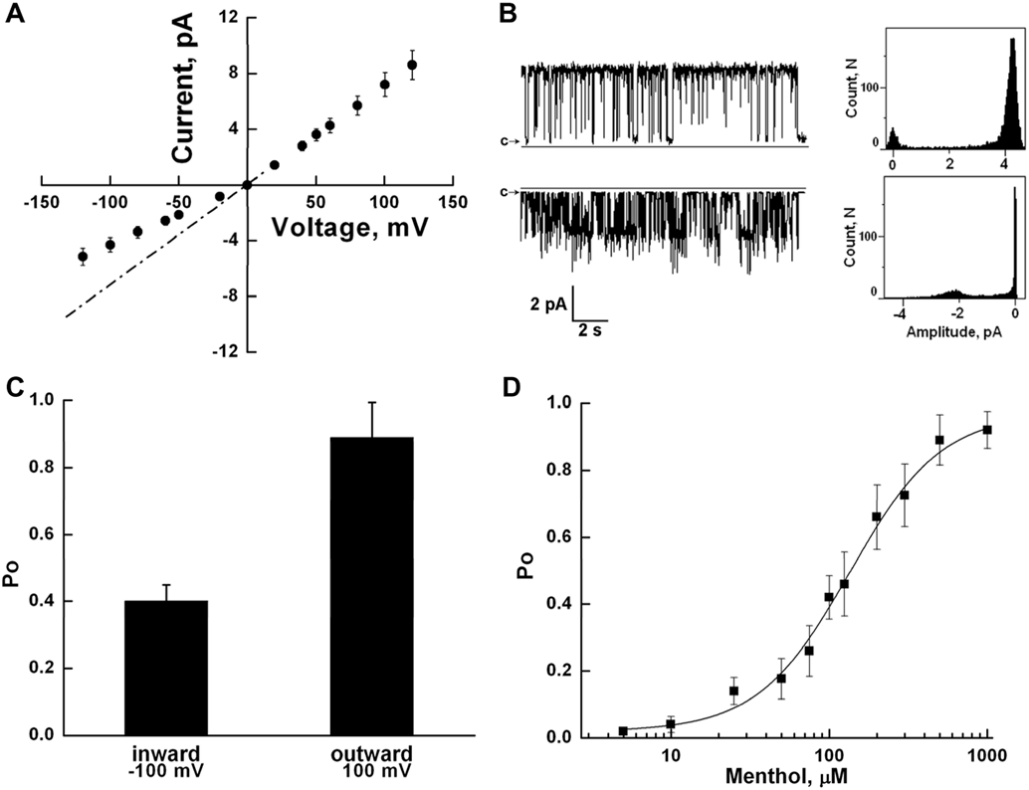
A. Representative current/voltage relationship of TRPM8: Channels were incorporated in planar lipid bilayers of synthetic POPC, POPE (3∶1) in the presence of diC_16_ PtdIns(4,5)P_2_. Experimental conditions are the same as described in the legend to Fig. 5. TRPM8 channels were stimulated with the application of 500 µM of menthol. The dashed line corresponds to the mean conductance of fully open channels, working in inward direction, this state is rarely observed due to the low open probability of this subconductance level. B: Representative current traces and all points' histograms of outward (upper) and inward (lower) currents of TRPM8 channels with clamping potentials were +60 mV and −60 mV, respectively. Experimental conditions are the same as in the legend to figure 6A. C: Open probability of TRPM8 channels operating in inward and outward directions measured at +100 mV and −100 mV. Data were analyzed from a total of 9 experiments. D: Menthol dose response of the open probability of TRPM8. Demonstrated P_o_ values were obtained at 100 mV. Data were analyzed from a total of 36 experimens.

After the conditions for obtaining the channels in lipid bilayers were established, we found that TRPM8 demonstrated different open probability and gating modes for current flowing in outward and inward directions. A single-channel current-voltage relationship, and open probabilities are presented in Fig. 6 (A–C). Channels were obtained in the presence of 1% diC_16_ PtdIns(4,5)P_2_ and 500 µM menthol. Outward currents exhibited mean slope conductance values of 72±12 pS, and P_o_ of ∼0.89 at 100 mV (n = 11, number of events analyzed = 2,811), and inward currents were observed in two conductance states with main conductance level of 42±6 pS and P_o_ of ∼0.4 (at −100 mV) and rarely detected burst openings of a subconductance state with mean conductance of 30±3 pS (P_o_≤0.001), which would step to the fully open magnitude (72 pS) of the channels (n = 10, number of events analyzed = 1,908). The observed value of the mean conductance (72 pS at 22°C) is similar to that previously reported [5], [42]. The orientation of the channels incorporated in the lipid bilayer was determined by outward rectification inherent to TRPM8, and poly-lysine block [12]. As previously shown, poly-lysine blocks TRPM8 currents from the cytoplasmic side of the channel. Consistent with this, 30 µg/ml of poly-lysine added to the bath solution did not significantly inhibit Ca^2+^ signals evoked by 500 µM menthol in cells expressing TRPM8 (data not shown).

**Figure 7:**
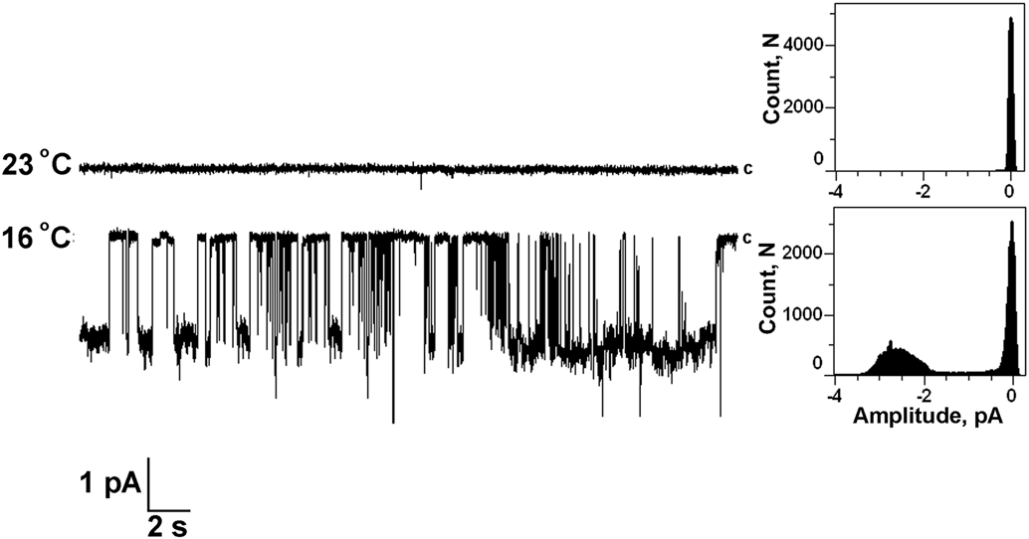
Activation of TRPM8 channels in Planar Lipid Bilayers by cold. Representative current traces of TRPM8 activated by lowering the temperature from 23 to 16°C in planar lipid bilayers: Channels were incorporated in planar lipid bilayers of synthetic POPC, POPE (3∶1) in presence of diC_16_ PtdIns(4,5)P_2_. Experimental conditions are the same as described in the legend to Fig. 6. Channels were inserted *cis* at 23°C and the temperature was then lowered to 16°C at ∼1 degree per min. Upper trace: TRPM8 activity at 23°C; lower trace: TRPM8 channel activity at 16°C (representative of 12 independent experiments). The temperature of the chambers was controlled by pyroelectric controller (see Experimental Procedures). The temperature in the *cis* bath (ground) was read directly using a thermoelectric junction thermometer, which also served as a point of reference for the pyroelectric controller. Data were filtered at 50 Hz. Clamping potential was −60 mV.

Next we investigated TRPM8 channel activity under various menthol concentrations and determined the menthol dose response on the single channel level (Fig. 6D). We found that menthol at different concentrations affects TRPM8 activity by mainly altering the open probability of the channel (Fig. 6D). In the figure 6D values of P_o_ observed at 100 mV were plotted against menthol concentrations, total 36 experiments were conducted and numbers of events analyzed for each menthol concentration were in a range of 400–1500. We also studied the cold sensitivity of TRPM8 reconstituted into planar lipid bilayers. Figure 7 demonstrates representative current traces of TRPM8 in planar lipid bilayers activated by lowering temperature from 23°C to 16°C. Total 12 independent experiments were conducted. These experiments confirm that the TRPM8 protein reconstituted into artificial lipidic bilayer resembles the properties of the native channel and can be successfully used for studies.

**Figure 8:**
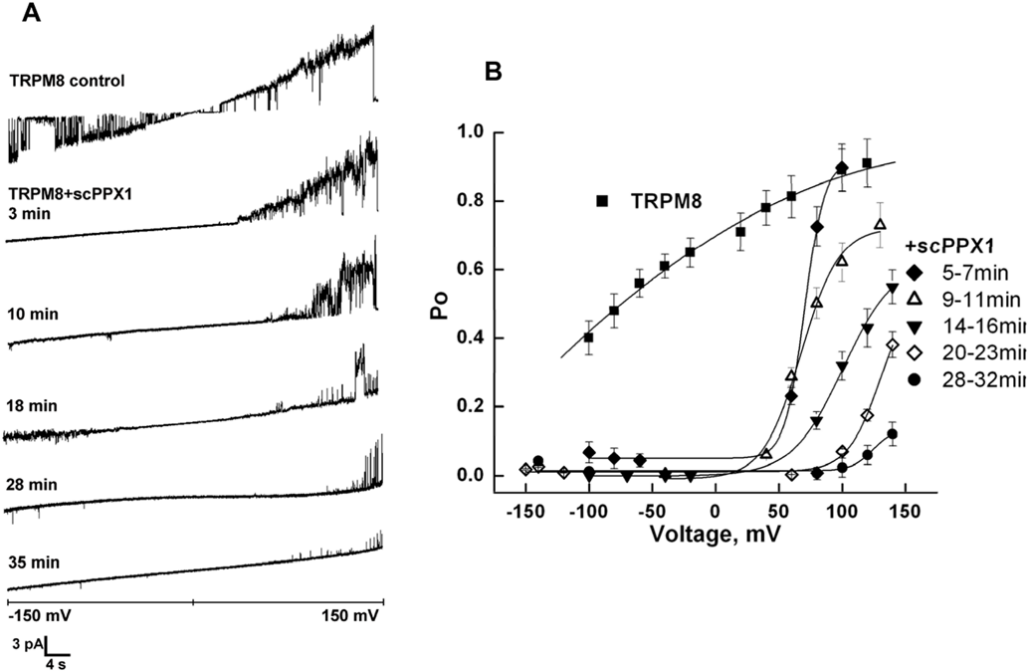
Voltage-dependence of TRPM8 before and after the treatment with scPPX1. A: Representative current traces recordings obtained at −150 +150 mV voltage ramps before and after the treatment with polyphosphatase in a time course at the beginning of 3^rd^, 10^th^, 18^th^, 28^th^ and 33^rd^ minutes. B: The changes in open probability obtained at different voltages in gap free recordings for TRPM8 alone (▪) or after the treatment with scPPX1 for the following intervals of time: 5–7 min (♦), 9–11 min (Δ), 14–16 min (▾), 20–23 min (◊), and 28–32 min (•). Data were analyzed from overall of 16 experiments.

**Figure 9:**
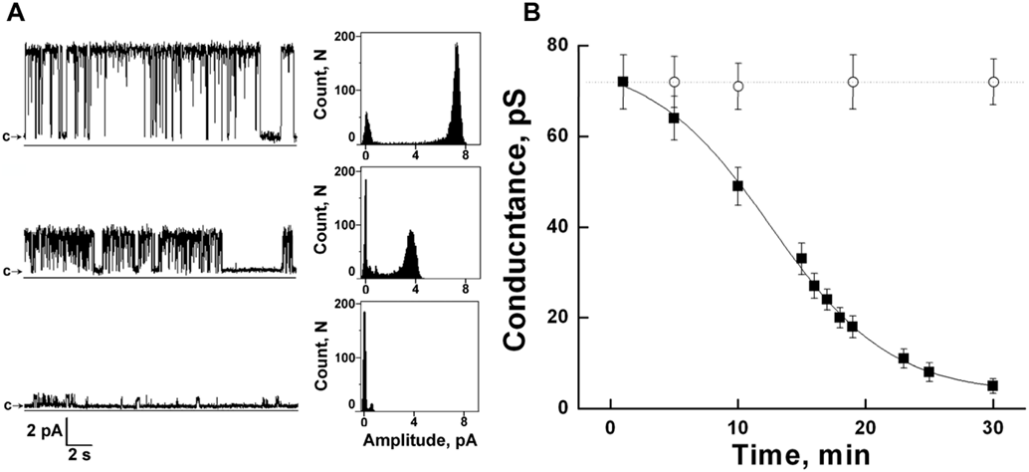
Reduction of TRPM8 channel conductance by exopolyphosphatase scPPX1. A: Representative single-channel current recordings of TRPM8 channels: upper traces – TRPM8 channels recordings before treatment with scPPX1; middle traces – TRPM8 channel recording 15 minutes later after the addition of scPPX1 (2 µg); lower traces – TRPM8 channel recordings after 30 minutes of addition of scPPX1. Clamping potential was +100 mV. Data were filtered at 50 Hz. B: Symbols (▪) (n = 5) correspond to the mean conductance values of scPPX1 treated TRPM8 channels, where 2 µM of scPPX1 were added to the internal side of the channel; (○) (n = 8) mean conductance of control, untreated channels. Experimental conditions are the same as described in the legend to Fig. 6.

Further, we examined the effect of scPPX1 on the single channel activity of TRPM8 in planar lipid bilayers (Fig. 8, 9). Channels were obtained in presence of 1% diC_16_ PtdIns(4,5)P_2_ and 500 µM menthol with subsequent addition of 2 µg/ml scPPX1. No change in channel activity was detected when scPPX1 was added to the external side of TRPM8. Conversely, addition of scPPX1 to the internal side resulted in the inhibition of TRPM8 currents by affecting both the open probability and conductance of the channel. We first analyzed the changes in the open probability of the channel upon the cleavage of polyP. We found that TRPM8 channel openings in inward direction were eliminated very rapidly (within 1–2 min) after the addition of scPPX1. In contrast, the open probability of the channel in outward direction exhibited much slower changes (up to 30 min). Figure 8A demonstrates current traces recordings obtained at −150 +150 mV voltage ramps before and after the treatment with scPPX1 in a time course at the beginning of 3^rd^, 10^th^, 18^th^, 28^th^ and 33^rd^ minutes. The statistics of the changes in open probability was obtained at different voltages in gap-free recordings for TRPM8 alone or after the treatment with scPPX1 for the following intervals of time: 5–7 min, 9–11 min, 14–16 min, 20–23 min, and 28–32 min (Fig. 8B). Overall 16 experiments were conducted and open probabilities values obtained for each voltage were derived from the analysis of at least 550–2636 events.

Next we analyzed the effect of scPPX1 on channel conductance. We found that, while TRPM8 inward currents were quickly eliminated by scPPX1, outward currents were gradually inhibited within 30 min (Fig. 9). The upper traces of representative currents and all points histogram in Figure 9A show TRPM8 activity before the application of scPPX1; the middle traces and histogram were obtained after 15 min of treatment with the enzyme; and lower traces and histogram show the channel activity after 30 min of scPPX1 addition. The reduction of TRPM8 channel conductance is demonstrated in Figure 9B (n = 5). The single-channel conductance approached zero upon cleavage of polyP with scPPX1. Channels not treated with scPPX1 displayed no change in conductance within the time of the experiments (n = 8).

## Discussion

The activity of TRPM8 channels is regulated by a plethora of factors including temperature, ligand binding, voltage, pH, etc. reflecting diverse stimuli and mechanisms of these regulators. In our study, we found that inorganic polyphosphate plays an essential role in determining the activity of TRPM8 channels. Furthermore, our data reveal that polyP is associated with TRPM8 in a supra-molecular complex.

Inorganic polyphosphate is present in all eukaryotic cells, however its function is not well defined [25], [26], [27]. Despite the abundance of polyP in all mammalian tissues, its evolutionary role and participation in many general physiological processes in the cell, polyphosphate is still a mysterious molecule, since little is known about the mammalian enzymes that control the levels of the polymer. A novel mammalian exopolyphosphatase has recently been identified: the DHH superfamily human protein h-prune, which shows high sequence homology to the known PPXs, was shown to be an efficient exopolyphosphatase [43]. However, unlike other PPXs, h-prune hydrolyses only short-chain polyP, but the long-chain polymers rather inhibit the activity of the enzyme. This leaves an open niche for the existence of another enzyme that would cleave long-chain polyP. On the other hand, endopolyphosphatase (PPN) activity was earlier found in several mammalian tissues by A. Kornberg's group [26]. The PPN activity was present in rat tissues, particularly in brain, heart, lung and kidney, but the attempts to identify the protein responsible for this activity have not been successful. Even less is known about mammalian polyP synthases. No mammalian homologs to the known polyP kinases (PPK) have been found in protein databases, and no enzymes comparable to PPK have been identified. Also, it was reported that in mammalian cells and tissues the synthesis of polyP from P_i_ bypasses the intracellular pools of Pi and ATP [27], suggesting that the synthesis of polyP in animals proceeds through a completely distinct enzymatic pathway, compared to one in bacteria and lower eukaryotes.

PolyP – a molecule of many functions – has frequently been shown as an active compound of several ion transporting systems, including some bacterial ion channels [33], [44], [45]. Recently, it has been reported by Kim and Cavanaugh that the presence of the polyanion is required for the regulation of the channel activity of one member of the mammalian TRP superfamily, TRPA1 [23]. The authors demonstrated using inside-out patches of HEK-293 cells expressing TRPA1 that short polyphosphates (2–65 residues) are required for sensitizing this channel to pungent chemicals and for preserving the protein in the functional conformation. The activity of TRPA1 channels was recovered in excised patches only in the presence of polyP and was diminished upon washing of polyP from the bath solution, which suggests weak interactions between polyP and TRPA1. Our own data show that polyP is also an important factor controlling channel activity of TRPM8, however the nature of these interactions is quite different from those observed by Kim and Cavanaugh. We found that polyP is co-purified with the TRPM8 protein in ensemble and removal of polyP from the protein can be achieved by its enzymatic digestion with scPPX1, indicating strong interaction between TRPM8 and polyP.

We first found the requirement of polyP for TRPM8 channel activity in whole-cell patch clamping of HEK-293 cells expressing TRPM8, where menthol-induced currents were inhibited upon hydrolysis of polyP by scPPX1. Polyphosphatase dialyzed via the patch pipette inhibited menthol-induced TRPM8 currents at −60 mV by 60% (Fig. 1A–C), while 80–90% inhibition was observed when scPPX1 was co-expressed with TRPM8 in HEK-293 cells (Fig. 1D–F, 2A–C). Enhanced inhibition of TRPM8 currents by scPPX1 in these experiments suggests that a longer exposure of the TRPM8 protein by expression of the enzyme ensures better access to polyP than in those experiments performed with acute scPPX1 treatment of TRPM8 via the patch pipette. The current/voltage relationship obtained in whole-cell patch clamp experiments revealed that inward TRPM8 currents exhibit a profound inhibition in the cells with co-expressed scPPX1 (∼83%), while outward currents are inhibited to a lesser extend (∼65%) (Fig. 1G–I).

Alternatively, we monitored calcium signals in F-11 neuronal cells expressing TRPM8 with/out scPPX1 (Fig. 2D–F). Similarly to HEK cells expression system, we found that Ca^2+^-entry was significantly inhibited in F-11 cells when TRPM8 was co-expressed together with polyphosphatase, which indicates that polyP plays an analogously important role on the TRPM8 activity in these cells as well. These results demonstrate a novel and significant contribution of polyP to TRPM8 channel activity.

The question of how polyP contributes to the regulation of TRPM8 channel activity motivated us to look at the biochemical and biophysical properties of TRPM8 in a reconstituted system. In experiments on TRPM8 purified from HEK-293 cells, we detected that polyP is associated with the protein (Fig. 4A). The assembly of polyphosphate with bacterial membrane proteins has been previously reported for a number of ion channels, where it is usually derived in a complex with the solvating polyester PHB [33], [44], [45]. PHB, possessing electron-donating oxygens closely spaced along a flexible backbone, is capable of solvating salts of hard cations; its amphiphilic nature allows it to penetrate hydrophobic regions inaccessible to water. Considering possible interactions of TRPM8 with the polymer, we further analyzed the protein for its association with PHB and indeed found that TRPM8 is associated with the polyester (Fig. 4B).

Addressing the question of the nature of interaction of the two polymers with the protein, we suggest that polyP possibly interacts with TRPM8 oligomers by ionic bonds as it is found in the association with TRPM8 tetramers. However, highly water soluble polyP might easily dissociate from the protein when it is converted to the monomeric form. PHB, in contrast to polyP, is insoluble in water and it is probably located in a hydrophobic region of TRPM8. Due to the strong interactions with the protein, which might include hydrophobic or even covalent bonds, PHB, unlike polyP, does not dissociate from the monomers of TRPM8 (Fig. 4B, lane 2). Complexes of polyP/PHB have been shown to form cation-selective channels in bacterial and mitochondrial membranes [30], [31], [32]. Moreover, these complexes have been identified in association with some ion channels including porins [33], [46].

An exciting model within these protein/polyP/PHB complexes has been represented by a potassium channel of *Streptomyces lividans* KcsA [44], [47]. Association of the polymers appears to contribute to the ion selectivity and gating properties of KcsA, and to determine its preference between mono- and divalent cations [34], [48].

It is conceivable that, in the case of the TRPM8 channel, similar formation of a polyP/PHB complex might take place. Alteration of the polymers associated with the protein could be a useful approach to demonstrate their function in the complex. However, due to the amphiphilic nature of PHB it is difficult to eliminate it from the native protein. For this reason, we concentrated our attention on the soluble component of the complex – polyP – and its enzymatic degradation by scPPX1.

In order to study the details of polyP participation in regulation of TRPM8 activity, we examined how scPPX1 modifies single channel activity of the purified TRPM8 reconstituted into planar lipid bilayer. Although, one distant homolog of TRPs, the polycystin-2 protein, that belongs to TRPP subfamily, has been shown to form functional channels in lipid bilayers [49], no TRP channels from the major subfamilies (TRPC, TRPV, TRPM) have previously been studied with this technique, to our knowledge. We first aimed to identify the conditions necessary to preserve the protein's stability and function during extraction, purification and subsequent incorporation into the artificial lipids. We found that it was critical to deliver TRPM8 to the final step of purification in its tetrameric form, since altering different conditions, such as ionic strength, osmotic pressure or detergents led to disintegration of tetramers into the lower assemblies, and ultimately to monomers. The stability of TRPM8 in tetrameric form was also important to prevent polyP dissociation. If such dissociation took place during purification, these TRPM8 proteins failed to form functional channels. After finding the optimal conditions for the purification, we attempted to detect TRPM8 channels after reconstitution into planar lipid bilayers. By testing many experimental conditions, we found that the TRPM8 channels in planar lipid bilayers can be activated by both menthol and cold only in the presence of PtdIns(4,5)P_2_ (Fig. 5, 7). This result was in agreement with previously reported data showing the requirement of PtdIns(4,5)P_2_ for the channel activity of TRPM8 [11], [12]. To our knowledge, our data provide the first demonstration of the dependence of mammalian ion channel activity on PtdIns(4,5)P_2_ in a reconstituted system. This is strong evidence that PtdIns(4,5)P_2_ regulates these channels through direct association, and not through an intermediary PtdIns(4,5)P_2_ binding protein.

When the appropriate conditions were established, we tested the effect of scPPX1 on the channel activity of TRPM8 and found that addition of the enzyme to the internal side of the channel was followed by drastic inhibition of the channel activity. This was based on a reduction in open probability and single-channel conductance to the point of complete loss of conduction (Fig. 8, 9). We detected that while the inward currents were eliminated very rapidly, the outward currents exhibited changes within 30 min of treatment with scPPX1. The kinetics of polyP cleavage by scPPX1 is dependent on the Mg^2+^ concentration, where Mg^2+^ is a cofactor for the enzyme activity [50]. In our studies, during exposure to the enzyme and polyP hydrolysis (Fig. 9B), inhibition followed a sigmoid time course, which may have resulted from both the Mg^2+^ concentration and accessibility of polyP from the protein.

Apparent voltage-dependence of the accessibility of polyP was also observed. PolyP was more vulnerable to scPPX1 when inside-positive voltages were first applied to provide an electrical driving force to favor an extension of polyP out from the protein on the cytoplasmic side. This important observation suggested a possible location of polyP in the inner cavity of channel conducting path and led us to look closely whether alternation of polyP from the channel has an effect on voltage-dependence of TRPM8. The voltage-dependence of TRPM8 has previously been reported by others [51], [52]. In our experiments we found that at certain conditions TRPM8 exhibits a slight voltage-dependence, which, however, undergoes significant changes upon the hydrolysis of polyP by scPPX1 (Fig. 8). These results demonstrate that polyP not only co-purifies with TRPM8 protein, but also modifies its function, and is thus directly involved in the regulation of TRPM8 channel activity.

The inhibition pattern, caused by scPPX1, suggests two distinct mechanisms: one resulted in a rapid decrease in channel openings observed in, physiologically relevant to TRPM8, inward currents, while the other is seen as a slow decline in conductance and channel openings detected in outward currents (Fig. 8, 9). The molecular mechanism of this regulation is elusive at this time and more studies are required for better understanding of the role of polyP in this channel/polymer complex. One possible role for polyP in the activity of TRPM8 might be to determine permeation and selectivity of the channel. Such a role of polyP has been shown for a potassium ion channel KcsA [34], [48], [53]. It is also not clear how scPPX1 treatment causes the reduction of single-channel conductance that we observe for TRPM8. It could be due to structural changes that may take place within the intracellular domains of TRPM8 upon removal of polyP. On the other hand, the reduced apparent single channel conductance could also be due to filtering of rapid, incompletely resolved gating transitions, or a “fast block” process, due to the uncovering of low affinity blocking sites of solutes by the removal of polyP.

In the context of the allosteric stimulatory and regulatory mechanisms that affect TRPM8, our study demonstrates that the TRPM8 protein coexists in a supramolecular complex with polyP and PHB, which alter its channel properties.

## Supporting Information

Figure S1 TRPM8 in HEK-293 cells with co-expressed scPPX1 Panel A: Images illustrate localization of myc-TRPM8 protein in control (left panel) and scPPX1 co-expressed (right panel) conditions. The protein was detected by FITC-Myc antibody. Arrows indicate localization of the myc-TRPM8 protein. Panel B: Western blot of TRPM8 probed with anti-Myc-IgG: Lane 1 – plasma membrane extracts of cells stably expressing TRPM8; Lane 2 – plasma membrane extracts of cells stably expressing TRPM8 and co-expressing scPPX1.
(2.08 MB TIF)

Methods S1 (0.03 MB DOC)
