# Supplementary figures and images for: Inorganic Polyphosphate Modulates TRPM8 Channels
Source: PLoS One. 2009 Apr 30;4(4):e5404. doi: 10.1371/journal.pone.0005404 (PMC2671608; doi:10.1371/journal.pone.0005404)

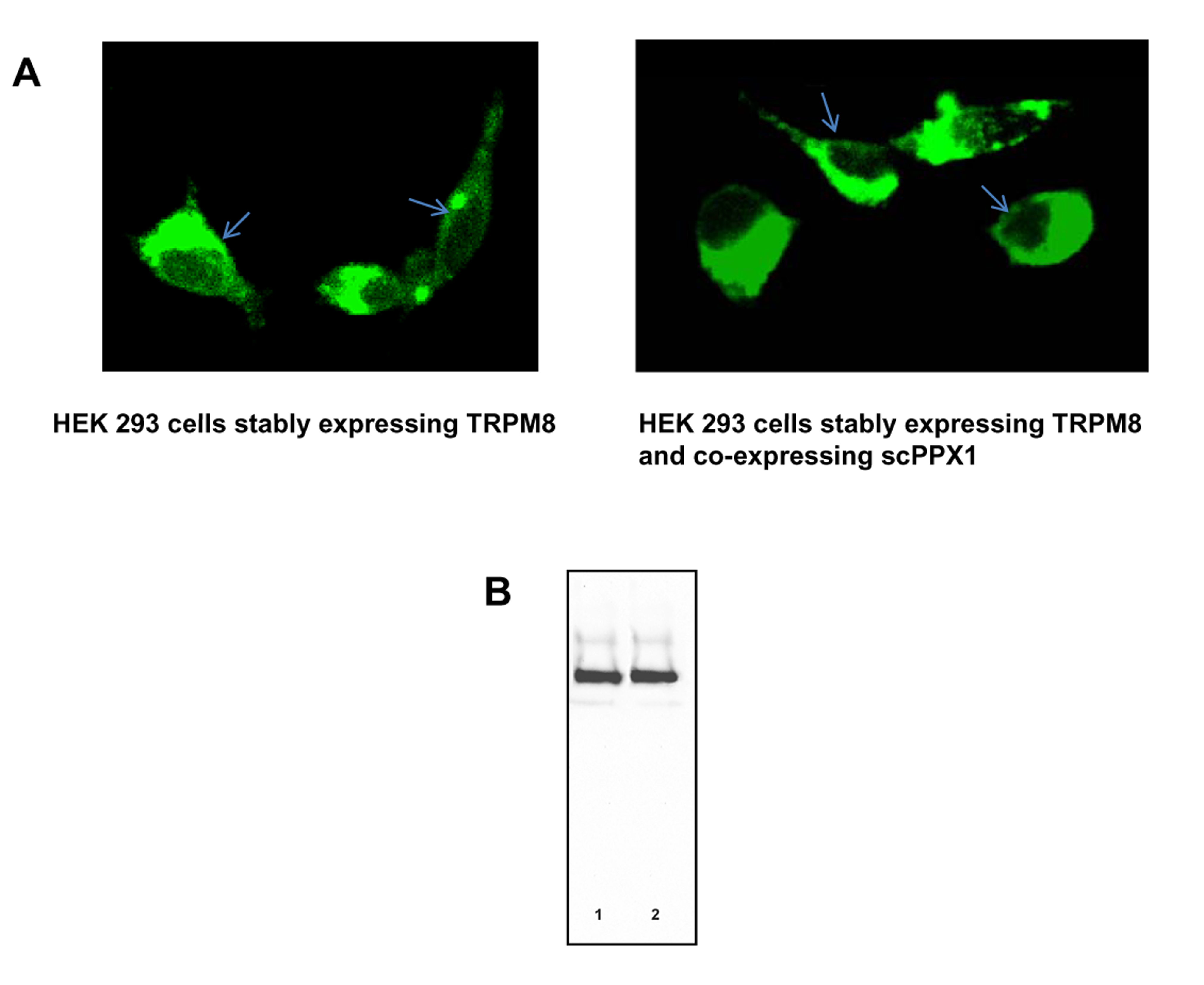

Supplement: Figure S1 — TRPM8 in HEK-293 cells with co-expressed scPPX1 Panel A: Images illustrate localization of myc-TRPM8 protein in control (left panel) and scPPX1 co-expressed (right panel) conditions. The protein was detected by FITC-Myc antibody. Arrows indicate localization of the myc-TRPM8 protein. Panel B: Western blot of TRPM8 probed with anti-Myc-IgG: Lane 1 – plasma membrane extracts of cells stably expressing TRPM8; Lane 2 – plasma membrane extracts of cells stably expressing TRPM8 and co-expressing scPPX1. (2.08 MB TIF) [file pone.0005404.s001.tif]
