# Supplementary material for: Inorganic Polyphosphate Modulates TRPM8 Channels
Source: PLoS One. 2009 Apr 30;4(4):e5404. doi: 10.1371/journal.pone.0005404 (PMC2671608; doi:10.1371/journal.pone.0005404)
Supplement: Methods S1 — (0.03 MB DOC) [file pone.0005404.s002.doc]

**Method:**

**Immunocytochemistry:** HEK-293 cells stably expressing myc-TRPM8 with or without transient expression of scPPX1 were grown on 25 mm round glass coverslips, were fixed with 2 ml of 4% paraformaldehyde in PBS at room temperature for 30 min. The cells were washed twice with PBS (2 ml) and treated with 2 ml of 100 mM glycine in PBS for 30 min. After washing with PBS twice, the cells were permeabilized with 300 µl of cold methanol at -20 ºC for 5 minutes and washed once with 2 ml PBS. The cells were then blocked with 5% goat serum for 1 hr at 37 ºC in an incubator followed by a wash with PBS and incubation with 300 µl of the FITC labeled anti-myc antibody (1:1000) rabbit) in PBS for another 1 hr at 37 ºC in an incubator. The cells were then washed three times (2 ml each) with PBS, washed once with distilled water, and mounted on a glass slide with mounting media, For immunohistochemical analysis cells were observed with a Zeiss (Oberkochen, Germany) LSM-510 confocal microscope, equipped with an argon laser (488 nm), in the Confocal Imaging Facility of the New Jersey Medical School.
